# Understanding patterns of adverse events after surgery and their impact on recovery

**DOI:** 10.1186/1745-6215-16-S2-O7

**Published:** 2015-11-16

**Authors:** Rachel L Nash, Barnaby C Reeves, Gianni D Angelini, Chris A Rogers

**Affiliations:** 1 Clinical Trials and Evaluation Unit, School of Clinical Sciences, University of Bristol, Bristol, UK; 2 Bristol Heart Institute, School of Clinical Sciences, University of Bristol, Bristol, UK

## Introduction

Clinical trial reports include data on adverse events experienced by study participants. Typically the frequency of each event is reported, but patterns of events occurring in one participant are rarely described. We explore associations between complications after surgery, and their impact on time-to-discharge as a first step to deriving an objective measure of recovery from surgery.

## Methods

The occurrences of different complications have been explored in a cohort of cardiac surgery patients, all of whom participated in an RCT. Multiple correspondence analysis (MCA) and latent class analysis (LCA) were used to identify associations and underlying “classes” of individuals based on complication profiles.

## Results

Data on 1453 patients from 6 clinical trials were collated. Sixteen complications were investigated; 44% of patients were complication-free, 31% experienced one complication, 14% two, and 16% three or more. Preliminary investigations showed that patients who experienced more severe complications (e.g. stroke) often had other, less severe, complications as well. Using LCA, three classes were identified; the class labelled ‘poor recovery’ had a high probability of serious complications (> 30%). As expected, post-operative stay was longest in patients assigned to the ‘poor’ recovery group (median 16 days, 95% CI [13,20] versus 9 [8,10] and 6 [6,6] in the ‘moderate’ and ‘good’ recovery groups, respectively).

## Conclusions

Many patients experience multiple complications. We have identified three classes with good face validity. The next step will be to investigate associations with routine data on vital signs (heart rate, temperature etc.), to identify which, if any can discriminate between recovery “classes”.

‘This work was carried out under an NIHR Research Methods Fellowship. The views and opinions expressed therein are those of the authors and do not necessarily reflect those of the HTA, NIHR, NHS or the Department of Health.’

